# Differences in the impact of leisure consumption spaces on urban residents’ life satisfaction: An empirical analysis based on social media big data

**DOI:** 10.1371/journal.pone.0347699

**Published:** 2026-04-30

**Authors:** Yaping Chen, Lu Zhang, Akot Deng

**Affiliations:** 1 School of Urban and Rural Construction, Zhejiang Guangsha Vocational and Technical University of Construction, Jinhua, China; 2 Guangdong University of Foreign Studies, School of English for International Business, Guangzhou, China; 3 Sudan University Science and Technology, Khartoum, Sudan; Tianjin University, CHINA

## Abstract

As residents’ demand for leisure consumption spaces continues to grow, the development of these spaces influences their perception of urban environments and life satisfaction. To examine how different urban leisure consumption spaces affect life satisfaction, we analyze service quality and life satisfaction using Dianping and Weibo Sign-in data through deep learning methods like Feature Tokenizer Transformer, then evaluate the relative importance of service quality’s impact. Results show that high service quality significantly enhances life satisfaction, while the quantity of spaces has negligible effect. Among different space types, Catering exerts the strongest influence on life satisfaction, followed by Entertainment, Personal care, Retail, and Sports, with regional and functional variations in these effects. This systematic study using multi-source big data and deep learning enriches media geography and spatial behavior theories while providing references for optimizing urban functional layout and public service policies.

## 1. Introduction

The urban functional layout is shifting from a production-centered approach to one that prioritizes living and services [1]. Urban space serves not only as a hub for economic activities but also as a vital setting for residents’ daily lives, social interactions, and leisure [2]. With the diversification of residents’ lifestyles, their demand for leisure consumption is growing rapidly. As a result, leisure consumption spaces have become a key indicator for assessing urban spatial vitality and livability.

Recent years have seen growing attention to research on livability and urban well-being, but existing literature mainly focuses on the impact of overall urban service functions or infrastructure on residents’ lives. Studies on the relationship between the development of leisure consumption spaces and residents’ life satisfaction remain relatively limited [3,4]. Therefore, in-depth research on the connection between the development of leisure consumption spaces and resident satisfaction not only helps enrich the theoretical framework of urban spatial social benefits but also provides empirical support for policymaking in urban residential space planning.

## 2. Literature review

Advances in information and communication technologies have transformed how humans access information and perceive space, reshaping the traditional logic of urban spatial structure centered on geographic location [5]. In this context, media geography offers a new perspective, emphasizing that individuals undergo changes in bodily perception, spatial behavior, and social practices through digital media engagement [6,7]. Social media platforms, map applications, and interest-based recommendation systems guide consumption behavior through algorithms, weakening traditional locational advantages to some extent. They enhance the visibility and clout of non-central areas, driving the reproduction and functional transformation of urban spaces [8]. Consequently, urban research has entered a behavior-driven phase of functional identification. On one hand, studies on intra-urban spaces have shifted from static land use analysis to dynamic tracking of human activity patterns [9,10]. On the other hand, big data has enabled a paradigm shift—from defining what urban space is to understanding how it is used [11,12]. Urban spatial research is increasingly being replaced by multidimensional spatial modeling methods that focus on individual movement trajectories, point-of-interest (POI) distribution, and sentiment analysis.

In recent years, with the deepening of urban renewal and the restructuring of residents’ lifestyles, urban leisure consumption spaces have become a key dimension for measuring urban livability and the balance of public services [13]. Compared to the earlier spatial division paradigm dominated by functional zoning, current research emphasizes consumption accessibility, sense of place, and opportunities for social interaction within residents’ living circles. These factors not only shape the distribution pattern of urban space, but also produce a profound impact on residents’ life satisfaction, sense of belonging, and sense of psychological safety. Specifically, the level of consumption accessibility determines the convenience degree that residents obtain leisure resources, which directly affects their daily leisure experience and life satisfaction [14]. Meanwhile, the sense of place makes residents produce emotional connections through spatial scene creation and cultural adaptation, which gradually condenses into a sense of belonging to the community and the city [15]. Furthermore, the environmental safety and service friendliness of leisure space can better build residents’ sense of psychological safety. These three factors intertwine with each other, which jointly constitute residents’ core subjective perception of the urban living environment [16]. Research shows urban leisure consumption spaces are shifting from single-center agglomeration to multi-center networked layouts. This structural transformation not only improves overall convenience of leisure consumption facilities, but more importantly enhances residents’ access to leisure resources across different neighborhoods, thereby influencing their satisfaction with living environments [17,18]. Whether leisure consumption spaces are embedded within residents’ daily living ranges has become a key indicator for measuring urban equity and well-being perception [19]. Studies reveal that micro-neighborhood commercial areas are gradually replacing large retail complexes, becoming frequent social hubs for community residents to gather, interact and regulate emotions [20]. Though small in scale, these micro-consumption spaces more effectively enhance residents’ sense of familiarity, security and satisfaction. Beyond physical accessibility and spatial distribution, the emotional regulation function of leisure consumption spaces is gaining attention. Non-consumption attributes like the allocation of blue-green spaces, openness, family-friendly amenities and cultural ambiance indirectly boost daily life pleasure and identity by shaping residents’ psychological and spatial emotions [21,22].

Traditional life satisfaction studies mainly rely on questionnaire surveys and structured interviews, emphasizing the explanatory power of objectively quantifiable indicators such as housing conditions, transportation convenience, income levels, and medical/educational resources [23,24]. However, recent studies increasingly show that residents’ satisfaction with urban life often does not strictly correspond to their material conditions, but is more influenced by spatial perception, neighborhood interaction, emotional support, and daily life experiences [25–27]. Especially in large cities, even with high socioeconomic status, residents may report low life satisfaction if their daily lives lack convenience, sense of belonging, and spatial comfort [28]. Therefore, subjective perception indicators are gradually being incorporated, including perceived accessibility of leisure resources, sense of security in public spaces, and availability of social support. This marks a paradigm shift from measurable environments to perceived environments [29,30]. In recent years, numerous studies have introduced sentiment analysis and text mining methods to conduct semantic recognition and emotion classification of residents’ unstructured texts on social platforms, review systems, and forum posts. These approaches utilize natural language processing techniques to extract information such as text sentiment polarity, emotion intensity, and keyword popularity, thereby reflecting residents’ real perception and emotional feedback towards specific spaces or urban life dimensions. Thereby, it realizes the three-dimensional depiction of space, time, and emotion of residents’ subjective well-being [31]. Specifically, natural language processing technology extracts the emotional polarity, intensity, and core keywords of texts. This accurately captures residents’ real experiences towards various leisure spaces [32]. Moreover, combining the geographic coordinates of texts locates the spatial positions that correspond to the perceptions. Meanwhile, matching timestamps grasps the temporal variation law of well-being. Then, emotional quantitative analysis supplements these steps. Ultimately, these methods realize the three-dimensional and dynamic depiction of residents’ subjective well-being [33].

With urban spatial research shifting from physical cities to perceived cities, the potential impact of urban leisure consumption spaces development on life satisfaction has gradually become an academic focus, as these spaces serve as important carriers of residents’ daily life experiences [29]. Studies generally agree that leisure consumption spaces indirectly improve residents’ quality of life by providing platforms for socialization and self-realization [2]. In terms of functional mechanisms, the social roles of leisure consumption spaces – such as providing gathering places, family-friendly amenities and cultural consumption opportunities – can strengthen neighborhood interactions and community belonging. It’s worth noting that more leisure consumption spaces don’t necessarily lead to better outcomes, as their effects largely depend on spatial distribution, service quality and integration with daily life [34,35]. Additionally, residents’ subjective perceptions of leisure space service quality often influence their life satisfaction more than objective quantity does. This shift prompts researchers to gradually move from studying spatial quantity to exploring spatial experience. Although existing studies have revealed correlations between leisure consumption space development and residents’ life satisfaction, most literature relies on questionnaire data and lacks comprehensive analysis combining both movement trajectories and sentiment data [36].

Based on the existing literature review, we aim to examine the relationship between the development level of urban leisure consumption spaces and residents’ life satisfaction through both individual movement trajectories data and sentiment analysis methods. This approach will provide practical empirical references for optimizing urban spaces and improving residents’ quality of life.

## 3. Theoretical hypotheses and research framework

### 3.1. Theoretical hypotheses

The theory of spatial production suggests that urban spaces emerge through human social practices and capital production. The construction of consumption spaces profoundly influences residents’ daily life quality and perception modes. The density, types, and distribution of consumption spaces constitute crucial components of urban spatial social production, affecting how residents experience space and daily life [37]. Environmental psychology suggests that individuals’ emotional experiences closely relate to the physical and perceptual characteristics of their surroundings. The aesthetic quality, safety, and functionality of spatial environments influence people’s psychological states and emotional expressions. Residents’ emotions around consumption spaces reflect their psychological responses to these environments, while the consumption spaces themselves serve as quantifiable representations of spatial perception experiences [38]. Urban quality of life and well-being theory suggests that quality of life depends not only on objective factors like income but also on subjective experiences such as transportation convenience, service accessibility, social opportunities, and cultural atmosphere. Good service facility provision helps improve residents’ well-being and subjective satisfaction. As a core component of urban subjective environment, the quality and diversity of leisure consumption facilities directly affect residents’ life satisfaction and emotional state [39,40]. Based on these fundamental theories, we propose the following research hypotheses:

H1: The service quality of leisure consumption facilities influences urban residents’ life satisfaction more significantly than the quantity of these facilities.

H2: Different types of leisure consumption spaces show significant spatial heterogeneity in their impact on residents’ life satisfaction.

### 3.2. Research framework

**Fig 1:**
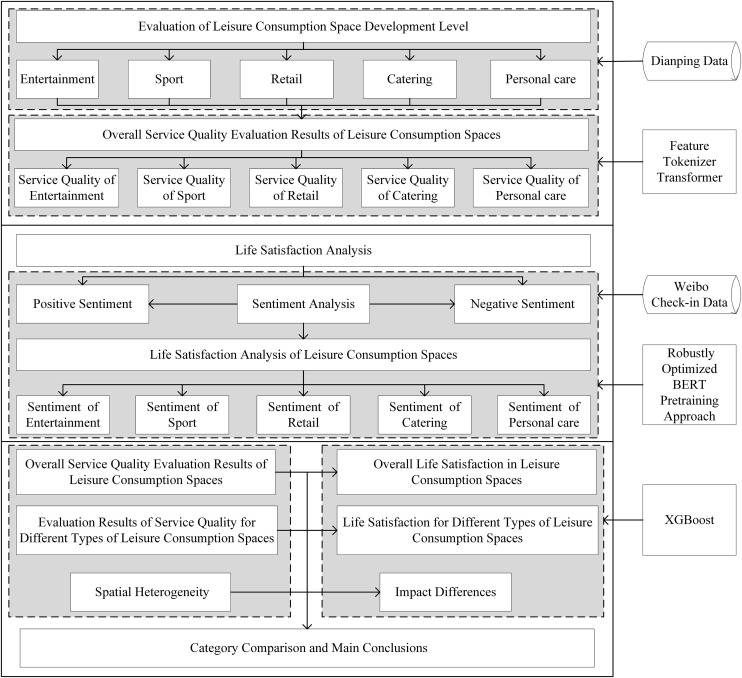
Workflow. (This figure systematically displays the overall technical route and analysis process of this study. Specifically, the whole process includes three major core stages: the service quality evaluation of leisure consumption space, the measurement of residents’ life satisfaction, and the analysis of influence relationships and relative importance. Furthermore, each stage progresses step by step and connects logically).

Based on our research hypotheses and specific research questions, we systematically organize the research framework. We divide the overall research process into three parts. The first part evaluates service quality of leisure consumption spaces. Using Dianping data, we categorize leisure consumption spaces into catering, entertainment, retail, personal care, and sports spaces. We employ Feature Tokenizer Transformer to assess service quality across different space types. The second part measures life satisfaction in leisure consumption spaces using Weibo sign-in data, applying Robustly Optimized BERT Pretraining Approach for sentiment analysis of sign-in texts. The third part uses XGBoost to analyze the relative importance of service quality impacts from different types of urban leisure consumption spaces on residents’ life satisfaction, followed by comparative discussion and conclusions. The detailed research workflow is shown in Fig 1.

## 4. Data and methods

### 4.1. Study data

Our study consists of two parts. The first part analyzes quality of different types of leisure consumption spaces. We use Dianping data for this research component. As China’s leading local lifestyle service platform, Dianping aggregates massive merchant information and user-generated content covering multiple leisure consumption categories, comprehensively reflecting the composition and distribution of different consumption facilities in cities [41]. Dianping data centers on user ratings and reviews, enabling service quality measurement of consumption spaces from actual consumer experiences, which enhances behavioral authenticity and user orientation in the analysis [42].

When exploring the relationship between the development of urban leisure consumption spaces and residents’ life satisfaction, it is essential to consider not only the service supply characteristics of consumption spaces themselves, but also residents’ usage behaviors and subjective preferences toward these spaces. Therefore, before introducing Sina Weibo sign-in data, this study first obtains data related to the service quality of leisure consumption spaces from the Dianping platform. Data was collected from January to December 2023, covering 287 prefecture-level and above cities in 31 provinces (autonomous regions and municipalities) of China. To ensure representativeness and to avoid sample bias caused by a large number of merchants and dense reviews in economically developed cities, the initial data screening ensures that the scale distribution of sample cities is consistent with that of the National Bureau of Statistics. The sample includes 22% from first-tier and new first-tier cities, 35% from second-tier cities, and 43% from third-tier and below cities. This distribution aligns with the actual distribution patterns of urban population and consumption activities. The original dataset contains a total of 42 million records. To improve data availability, we conduct systematic data cleaning and standardization processing on the original data. The first step is duplicate value removal. Specifically, we compare merchant names, addresses, and latitude and longitude (three elements) through the Jaccard similarity algorithm. If the similarity is greater than or equal to 0.8, we judge the record as a duplicate merchant record and remove it. This solves the problem of cross-industry registration or information redundancy. The second step is missing value processing. We comprehensively search key fields such as ratings, review counts, and geographic locations. When a single record misses more than 2 core fields or the missing rate of a certain core field in the sample city is greater than 30%, we remove this record or the corresponding category record of the city. This ensures the integrity of the analysis variables. The third step is outlier detection and removal. We adopt the box plot method. We identify the values exceeding Q3 + 1.5IQR or falling below Q1-1.5IQR in each rating dimension (taste, environment, service) and the review counts as extreme outliers. Furthermore, we mainly exclude the records with more than 100,000 reviews in a single month or scores less than or equal to 1 point or scores greater than or equal to 5 points and fewer than 10 reviews. In this way, we remove distorted data such as click-farming and robot reviews. The fourth step is category standardization and classification sorting. We uniformly name and classify merchant categories according to the *Classification and Coding Specification for Urban Consumption Space in China*. We merge more than 200 detailed subcategories into five core categories: catering, entertainment, shopping, healthcare, and sports. This guarantees the consistency and comparability between analysis dimensions. Finally, the last step is structural bias control and weighting correction. Given that urban catering merchants account for a very high proportion in the review data (the original proportion reaches 62%), this may cause structural bias. Therefore, we introduce the city tier weighting (first-tier/ new first-tier equals to 0.22, second-tier equals to 0.35, third-tier and below equals to 0.43) and industry category weighting (calculating weights based on the proportion of residents’ leisure consumption expenditure in each city) strategies. We balance and adjust the sample contribution degree of different regions and categories through the weighted average method. This more reasonably reflects the overall leisure consumption space quality of the city. After these processes, we finally retain a total of 16,282,573 valid records.

When examining the relationship between urban leisure consumption space development and residents’ life satisfaction, besides considering service provision characteristics of the consumption spaces themselves, residents’ usage behaviors and subjective preferences also hold significant research value. Therefore, this study incorporates Sina Weibo sign-in data to characterize urban residents’ spatial usage patterns and subjective satisfaction trends from actual user experience behaviors.

Weibo sign-in data represents users’ voluntary spatial behavior records actively published via mobile devices at specific locations These sign-ins contain user-initiated expressions with spatial-emotional connections, showing higher expressiveness as users typically sign in at pleasant, significant or emotionally valuable locations [43]. Moreover, Weibo sign-ins record precise timestamps, locations and user comments, possessing strong temporal continuity and social semantic readability [44].

**Fig 2:**
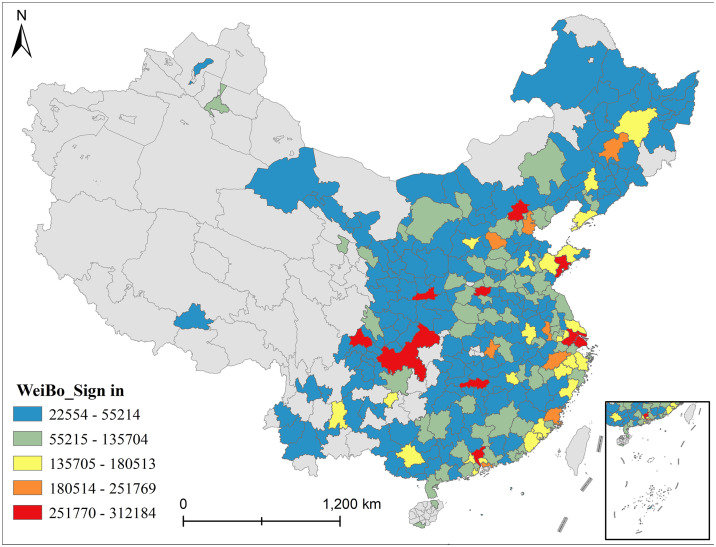
Spatial distribution of Weibo sign-in data. (This figure takes the form of a geospatial distribution map. It shows the spatial distribution characteristics of the valid Weibo sign-in data after cleaning in this study. Specifically, these data cover 287 prefecture-level and above cities across 31 provinces (autonomous regions and municipalities) nationwide. Furthermore, the figure uses color depth and point density to represent the quantity distribution of the sign-in data).

This study obtains geotagged public sign-in data from Sina Weibo’s API for the period of January to December 2023. The geographical coverage aligns with the Dianping dataset, encompassing 287 prefecture-level and higher cities across China’s 31 provincial divisions, ensuring spatial comparability between the two datasets. To address representativeness, the data undergoes regional demographic calibration alongside subsequent filtering for invalid records to ensure rationality. Since Weibo’s core user base predominantly consists of middle-to-high income individuals aged 18–45 (representing 78% of users), cities with elderly populations exceeding 15% received an additional 5% sample weighting to partially compensate for underrepresentation of older and lower-income demographics. The city-tier distribution mirrors the Dianping sample, maintaining consistency within the study’s focus on major urban areas. The final dataset comprises approximately 20 million raw records. To ensure the data effectively reflect residents’ life satisfaction, this study establishes specific data filtering and semantic discrimination mechanisms. First, we conduct spatial semantic matching and classification sorting. Based on the POI information of sign-in locations, we divide them into leisure consumption space categories closely related to residents’ daily lives through a geocoding matching algorithm. Specifically, these categories include catering, shopping, cultural entertainment, sports, and healthcare. Meanwhile, we remove sign-in points of non-leisure spaces such as government affairs, industry, and transportation. Second, we filter texts related to life satisfaction. We utilize natural language processing technology to conduct semantic analysis on the texts attached to the sign-ins. Then, we construct an emotion keyword library for life satisfaction (including 218 positive emotion words and 96 satisfaction representation phrases). If the texts match more than 1 positive emotion word or representation phrase and contain no negative emotion words, we include these records into the valid sample. Furthermore, we remove texts with no emotional expression or purely functional descriptions. Third, we remove fake and abnormal sign-ins. Through spatial-temporal sequence analysis, we detect records where the same user has more than 3 cross-city sign-ins within 24 hours or more than 5 multi-point sign-ins in the same city within 1 hour. This step excludes virtual positioning and chart-manipulation behaviors. At the same time, we remove robot account data with no geographic coordinates or a single-account monthly sign-in frequency more than 100 records. After the above cleaning and filtering processes, we finally retain about 5,319,234 valid sign-in records that relate to residents’ daily lives and have positive emotional expressions (see Fig 2). To further verify the robustness of Weibo sign-in data as a proxy variable for life satisfaction, we randomly extract samples from 30 cities of different tiers. Subsequently, we adopt Pearson correlation analysis to compare the consistency trend between sign-in density and Dianping satisfaction scores under different city sizes (correlation coefficient r equals to 0.78, p less than 0.01). Ultimately, this further supports the rationality of its use in this study.

### 4.2. Methods

#### 4.2.1. Feature tokenizer transformer.

In the task of evaluating the service quality of urban leisure consumption spaces, traditional machine learning models (such as Random Forest), Graph Neural Networks, and basic Transformer models are common candidate solutions. However, they all lack sufficient adaptability to the scenario of this study. Furthermore, they have significant limitations in aspects like robustness and feature processing capability [45]. Specifically, Random Forest achieves prediction through the integration of multiple decision trees. Its mechanism relies on feature splitting, so it cannot capture the interaction effects between dimensions like taste scores and environment scores. Moreover, it has a weak ability to process the mixed features of discrete scores (star ratings) and continuous indicators (positive review rates). In the pre-experiment of this study, its service quality evaluation shows an MAE of 0.62 and an RMSE of 0.85. Additionally, it has poor noise resistance. Under 5% to 10% random noise, its evaluation accuracy drops by 12.5%. When samples are sparse, its MAE increases by 0.21. Also, its cross-city evaluation generalization accuracy is only about 60%. Therefore, it struggles to adapt to the evaluation needs of leisure consumption spaces with multi-dimensional and heterogeneous features. Meanwhile, Graph Neural Networks can model spatial associations through the topological relationships of nodes and edges. However, they overly rely on spatial proximity [46]. They ignore the service details of a single venue and have a weak encoding ability for non-spatial features like service attitude in user reviews. In the pre-experiment, their evaluation accuracy is only 68.3%, and under 5% to 10% random noise, this accuracy drops by 9.8%. They only fit data analysis dominated by spatial features and fail to accurately reflect the core service quality of leisure spaces [47] Finally, basic Transformer models possess a global attention mechanism and can capture feature interaction effects. But they do not design an exclusive encoding layer for the differentiated features of multiple types of spaces. They indiscriminately weight the type-exclusive features of spaces like catering and entertainment. In the pre-experiment, their inter-category evaluation confusion reaches 31.7%. When samples are sparse, their MAE increases by 0.18, and their cross-city evaluation accuracy is about 72%. Thus, they easily cause confusion in the service quality evaluation of different leisure spaces.

Therefore, this study selects the Feature Tokenizer Transformer (FT-Transformer) to conduct the service quality evaluation of leisure consumption spaces. This model significantly outperforms the above existing methods in evaluation accuracy, robustness, and scenario adaptability [47]. Moreover, its core advantages and robustness features highly fit the research scenario of this study. This scenario involves Dianping big data and multi-city, multi-type leisure spaces. Firstly, it uniformly converts discrete scores and continuous indicators into low-dimensional vectors through a feature embedding layer. This mechanism perfectly solves the difficulty of processing heterogeneous mixed features [48]. Furthermore, its noise resistance is outstanding. Under 5% to 10% random noise, its evaluation accuracy only drops by 2.1%. This performance far exceeds existing methods. Secondly, it equips a multi-head self-attention mechanism. It accurately captures the interaction effects of multi-dimensional scores and restores the comprehensive perception logic of service quality [49]. Meanwhile, it combines an industry category weighting strategy to conduct differentiated learning on the feature weights of different leisure spaces. Also, it has low sample sensitivity. In the case of sparse sample sizes in underdeveloped western cities, its evaluation MAE only increases by 0.03. Thus, it solves the evaluation deviation problem that stems from insufficient sample sizes of leisure consumption data in small and medium-sized cities [50]. Thirdly, its lightweight model structure significantly reduces computational costs. It adapts to the analysis of tens of millions of Dianping big data. Simultaneously, it achieves overfitting control through the Dropout mechanism and Early Stopping. Therefore, its generalization ability is extremely strong [51]. In the cross-city service quality evaluation, its accuracy remains above 85%. Consequently, it perfectly adapts to the research needs of this study, and these needs cover cities of different levels.

The overall mapping process of FT-Transformer can be expressed as:


$$\hat{y}=f_{\theta }(x)=W_{\text{0}}\cdot Transformer(Z)+b_{\text{0}}$$

Where, θ denotes all learnable parameters of the model, including the mapping parameters of the feature embedding layer, the self-attention parameters in the Transformer encoder layers, and the weight $W_{\text{0}}$ and bias $b_{\text{0}}$ of the linear output layer. $x\in R^{n}$ represents the input *n*-dimensional rating feature vector (including star ratings, taste scores, number of positive reviews, etc.). $\text{Z}=\{{\text{z}}_{\text{1}},{\text{z}}_{\text{2}},,,,,,{\text{z}}_{\text{n}}\}$ denotes the feature representations after linear mapping or embedding. Z=Embedding(x), where Embedding(·) is the embedding function. It converts each dimension of the discrete or continuous features in *x* into vectors of uniform dimensionality, achieving nonlinear feature encoding and dimensionality reduction, thereby providing suitable input for the subsequent global feature extraction in the Transformer layer. Transformer(Z) indicates the global feature representations processed through multiple self-attention layers. $W_{\text{0}}$ and ${\text{b}}_{\text{0}}$ are parameters of the linear output layer, with the final output $\hat{y}$ being the model’s predicted composite score.

Within the Transformer module, the attention mechanism at each layer can be formally expressed as:


$$Attention(Q,K,V)=softmax(\frac{QK^{T}}{\sqrt{d}})V$$

Where, Q,K,V represent the query, key, and value vectors transformed from input features, with d denoting the embedding dimension. The multi-head attention mechanism enables the model to simultaneously learn multiple distinct attention patterns, thereby more comprehensively modeling the complex relationships among rating features. For this study, our implemented FT-Transformer model architecture consists of one feature embedding layer, three Transformer encoder layers, and one output layer. The input layer receives multi-dimensional rating features, with each feature dimension mapped to vector representations of uniform dimensionality through embedding mechanisms. We introduce Dropout in the Transformer layers with a rate of 0.1 to prevent overfitting, and employ Early Stopping during training – terminating when the validation loss fails to decrease for several consecutive epochs. The model training objective is to minimize the following loss function:


$$\Gamma =\frac{\text{1}}{n}\sum_{i=\text{1}}^{N}(y_{i}-\hat{y}{)}^{\text{2}}$$

Where, $y_{i}$ is the true rating of the *i*-th sample, $\hat{y}$ is the model’s predicted value, and *n* is the total number of samples.

#### 4.2.2. Robustly optimized BERT pretraining approach (RoBERTa).

In the task of sentiment analysis of residents’ life satisfaction, Naive Bayes, LSTM, and basic BERT models are mainstream candidate solutions. However, they all struggle to adapt to the core features of Weibo sign-in data texts, which include short texts, strong spatial associations, and fragmented sentiments. Furthermore, they have obvious deficiencies in aspects like robustness and semantic recognition capability [52]. Specifically, Naive Bayes achieves sentiment classification based on word frequency statistics. Its bag-of-words model cannot capture textual semantic associations. Its recognition accuracy for exclusive sentiment words of leisure spaces, such as “deserted” and “poor service,” is only 59.2%. In the pre-experiment of this study, its sentiment classification F1 score is 0.61. Additionally, it has extremely poor noise resistance. When the text contains noise like meaningless auxiliary words and internet slang, its F1 score drops by 15.2%. Facing short texts of 10–15 characters, its recognition accuracy is only 58.3%. Also, its cross-spatial type sentiment analysis F1 score is about 55%. Therefore, it is far below the needs of this study. Meanwhile, although LSTM can process text sequence relationships, it has the problem of insufficient capture of long-distance dependencies [53]. Facing short texts with an average length of only 12.8 characters in Weibo sign-in data, it struggles to mine the continuous recognition of leisure spaces. In the pre-experiment, its sentiment tendency misjudgment rate reaches 28.9%. Under text noise, its F1 score drops by 10.5%. Furthermore, its classification accuracy for sparse samples without sentiment descriptions is only 45%. Thus, it fails to adapt to the sentiment analysis scenario of Weibo short texts [54]. Finally, although basic BERT models improve semantic understanding capability through a bidirectional attention mechanism, they do not optimize for spatial short texts. They lack sufficient embedding representation for space-exclusive vocabulary. Moreover, they cannot output the sentiment difference results of different spatial types. In the pre-experiment, their spatial type sentiment classification accuracy is 72.5%. In sparse samples without descriptions, their classification accuracy is only 56%. Also, their cross-spatial type sentiment analysis F1 score is about 75%. Consequently, they struggle to meet the differentiated sentiment analysis needs of multi-type leisure spaces in this study.

Therefore, this study selects the Robustly Optimized BERT Pretraining Approach (RoBERTa) to conduct the sentiment analysis of residents’ life satisfaction. As an optimized version of BERT [55], this model significantly outperforms the above existing methods in sentiment recognition accuracy, robustness, and scenario adaptability. Moreover, its core advantages and robustness features perfectly adapt to the features of Weibo sign-in data and the needs of this study [56]. Firstly, it greatly improves the capability of semantic understanding and mining of short texts through dynamic masking and longer training sequence optimization [57]. Its recognition accuracy for Weibo short texts of 10–15 characters reaches 90.5%. At the same time, it increases the recognition accuracy of exclusive sentiment words of leisure spaces to 89.7%. Additionally, its noise resistance is outstanding. When the text contains noise, its sentiment classification F1 score only drops by 1.8%. This performance far exceeds existing methods. Secondly, it supports the introduction of POI types as context supplementary embeddings. It achieves the fusion encoding of spatial POIs and sentiment texts. Thus, it solves the association problem between sentiment and space [58]. It raises the spatial type sentiment classification accuracy to 88.3%. Furthermore, it has an extremely strong adaptation capability to data sparsity. In samples missing sentiment texts, it still achieves a 78% sentiment classification accuracy. Thirdly, its fine-tuning efficiency on small-scale annotated data is 30% higher than basic BERT. It adapts to the analysis needs of social media big data, and its generalization ability is stronger [59]. In the cross-spatial type sentiment analysis, its F1 score remains above 86%. Fourthly, this study adopts the pre-trained chinese-roberta-wwm-ext model. It conducts more accurate vocabulary embedding for Chinese online short texts. It can effectively recognize exclusive colloquial expressions of leisure consumption, such as “high cost-performance” and “stepping on a mine”. This is a core advantage that existing methods fail to achieve. Finally, in the pre-experiment of this study, RoBERTa shows an accuracy of 91.2% and an F1 score of 0.90 in the sentiment classification task of Weibo sign-in texts. All its performance indicators significantly outperform the above baseline models.

The overall computational process of the RoBERTa model can be formalized as:


$$\hat{y}=Softmax(W_{o}\cdot h_{\left[CLS\right]}+b_{\text{0}})$$

The input Weibo text forms an input sequence $\text{x}=\{{\text{x}}_{\text{1}},{\text{x}}_{\text{2}},,,,,,{\text{x}}_{\text{n}}\}$ after tokenization and embedding, and generates corresponding contextual representations $\text{H}=\{{\text{h}}_{\text{1}},{\text{h}}_{\text{2}},,,,,,{\text{h}}_{\text{n}}\}$ through the RoBERTa encoder. Here $h_{\left[CLS\right]}$ is the vector of the [CLS] token at the beginning of the sentence, serving as the global semantic representation of the entire text. $W_{o}$ and $b_{\text{0}}$ are the weight and bias parameters of the output layer. Finally, the model outputs probabilities for each sentiment category (such as positive, negative, neutral) through the softmax layer, thereby completing the sentiment classification task.

In this study, we use the pre-trained chinese-roberta-wwm-ext model and fine-tune it on the Weibo sentiment dataset. The model input consists of tokenized Weibo text sequences with a maximum length of 128 and an embedding dimension of 768. The Transformer encoder contains 12 layers, each with 12 attention heads and feed-forward networks, using residual connections and LayerNorm for stable modeling. During model training, we employ the cross-entropy loss function defined as follows:


$$\Upsilon =-\frac{\text{1}}{N}\sum_{i=\text{1}}^{N}\sum_{C=\text{1}}^{C}y_{ic}log({\hat{y}}_{ic})$$

Where, *N* is the number of samples, *C* is the number of categories, $y_{ic}$ is the true sentiment label of sample *i*, and ${\hat{y}}_{ic}$ shows the model’s predicted probability distribution.

#### 4.2.3. The XGBoost model.

In the importance recognition task of the impact of different leisure consumption spaces on life satisfaction, multiple linear regression, LightGBM, and random forest are common candidate solutions. However, they all struggle to meet the core needs of this study for nonlinear relationship capture and precise multivariable distinction [60]. Moreover, they have obvious limitations in aspects like model robustness and feature recognition accuracy [61]. Specifically, multiple linear regression assumes a linear relationship among variables. It fails to restore nonlinear rules, such as the diminishing marginal effect on life satisfaction after service quality improves to a certain threshold. Furthermore, it cannot process the interactive influence between the service quality of dining spaces and the service quality of entertainment spaces. In the pre-experiment of this study, its model goodness-of-fit R² is 0.58. Also, its feature importance recognition accuracy is only 65.3%. Therefore, it struggles to adapt to the complex relationship between leisure consumption spaces and life satisfaction [62]. Meanwhile, although LightGBM is a gradient boosting tree model, its histogram-based feature splitting mechanism easily confuses the impact of quantity increase and quality improvement on life satisfaction. In the pre-experiment, its feature importance discrimination confusion degree for “service quality” and “facility quantity” reaches 29.6%. Consequently, it fails to accurately distinguish core influencing factors [63]. Finally, although random forest can process nonlinear relationships, its calculation of feature importance during the ensemble process cannot quantify the contribution differences of different leisure space types. In addition, it lacks sufficient redundant processing capability for high-dimensional features. This situation leads to a decline in model interpretability [64]. In the pre-experiment, its standard deviation of feature importance distinction among types reaches 0.18. Thus, it struggles to accurately reveal the impact differences of different types of leisure spaces.

Therefore, this study selects the XGBoost model to analyze the relative importance of the impact of the quantity and quality of different types of leisure consumption spaces on urban residents’ life satisfaction. Furthermore, this model significantly outperforms the above existing methods in feature processing, relationship modeling, robustness, and scenario adaptability [65]. Its core advantages highly fit the research needs of this study. Firstly, the model can effectively process high-dimensional leisure consumption feature data. It reduces feature redundancy through column sampling and regularization strategies. Even in high-dimensional data, it still maintains stable modeling effects. Thus, it adapts to the multi-dimensional and multi-type feature variable settings of this study [66]. Secondly, it has strong nonlinear modeling capability. It accurately captures complex interactive effects among different feature variables through gradient boosting and second-order Taylor expansion. It can effectively restore the nonlinear relationship between leisure consumption spaces and life satisfaction. Therefore, it solves the problem that existing methods fail to capture marginal effect changes and interactive influences [67] Thirdly, the model has extremely strong robustness. It shows good adaptability when facing problems like missing data and heteroscedasticity. It does not need additional complex data preprocessing. Consequently, it adapts to the analysis scenario after the fusion of multi-source big data [68] Fourthly, it can accurately quantify the importance contribution of each feature by calculating the gain value (Gain) of each feature. It can effectively distinguish the impact differences between facility quantity and service quality, and among different types of leisure consumption spaces [69]. Its standard deviation of feature importance distinction among types is only 0.07. Its feature importance recognition accuracy reaches 90.7%. Moreover, its model goodness-of-fit R² is 0.82, which far outperforms existing methods. Thus, it can accurately reveal the relative importance of different types of consumption spaces in urban residents’ life satisfaction.

Objective Function: The XGBoost optimization objective function consists of two components – a loss function and a regularization term. The loss function measures the error between predicted values and actual values, while the regularization term controls model complexity to prevent overfitting.


$$Obj(\theta )=\sum_{i=\text{1}}^{N}L(y_{i},{\hat{y}}_{i})+\sum_{k=\text{1}}^{K}\Omega (f_{k})$$

Where, *N* represents the total number of samples used in training the XGBoost model, specifically the valid sample size for analyzing the relationship between leisure consumption spaces and residents’ life satisfaction. *K* denotes the total number of decision trees integrated into the XGBoost model, while θ represents all learnable parameters in the XGBoost model. $L(y_{i},{\hat{y}}_{i})$ is the loss function, $y_{i}$ and ${\hat{y}}_{i}$ represent the true value and predicted value of the *i*-th sample respectively. $\Omega (f_{k})$ serves as the regularization term that controls model complexity. The regularization term is defined as:


$$\Omega (f)=\gamma T+\frac{\text{1}}{\text{2}}\lambda {\left\|w\right\|}^{\text{2}}$$

Where, *T* is the number of tree nodes, γ and λ are tuning parameters, and *w* denotes the leaf node weights.

In each iteration, XGBoost fits a new decision tree to minimize the current model’s residuals. Specifically, the weight $w_{j}$ of each leaf node is determined by minimizing the following objective function:


$$w_{j}=-\frac{\sum_{i\in I_{j}}\vartheta_{\hat{y}}L(y_{i},{\hat{y}}_{i})}{\sum_{i\in I_{j}}\overset{\text{2}}{\underset{\hat{y}}{\vartheta }}L\left(y_{i},{\hat{y}}_{i}\right)+\lambda }$$

Where, $\vartheta_{\hat{y}}L$ and $\overset{\text{2}}{\underset{\hat{y}}{\vartheta }}L$ represent the gradient and second derivative of the loss function respectively, and $I_{j}$ denotes the sample set contained in leaf node *j*. Overall, the XGBoost model effectively handles complex relationships between different types of leisure consumption spaces and life satisfaction, revealing their relative importance in urban residents’ life satisfaction.

## 5. Results

### 5.1. The service quality of leisure consumption space

**Fig 3:**
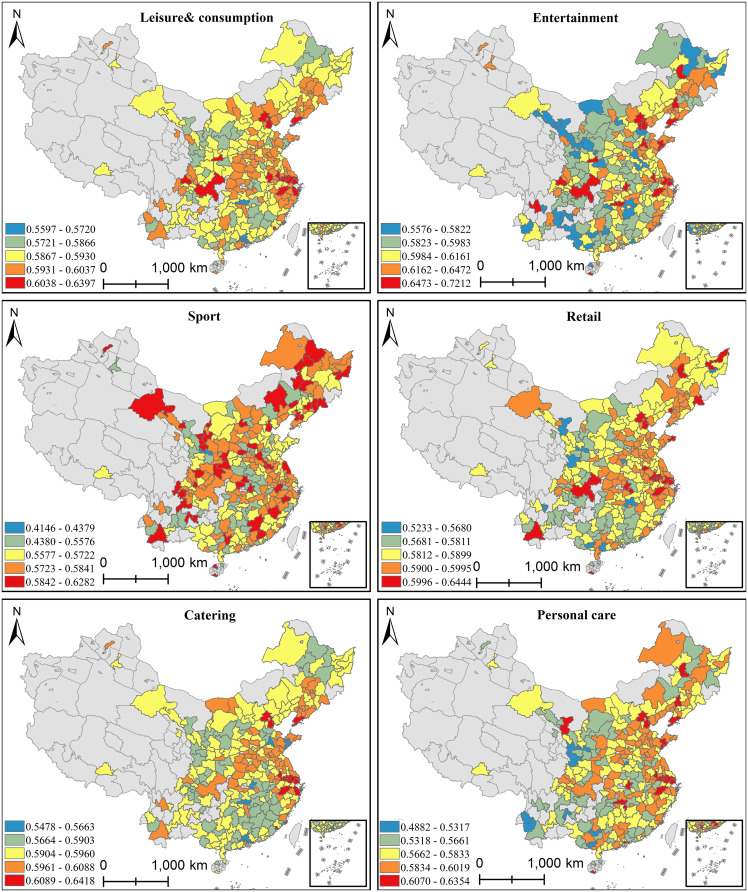
Service quality evaluation results of different types of leisure consumption spaces. (This figure is a series of spatial distribution maps. Specifically, it includes five sub-maps of dining, entertainment, shopping, healthcare, and sports. Furthermore, it uniformly adopts a service quality rating scale of 0 to 1. In addition, it characterizes the level of service quality of various leisure consumption spaces in different cities with color gradients. Here, deeper colors represent higher service quality. Ultimately, it systematically shows the national spatial pattern and type differences of the service quality of the five major categories of leisure consumption spaces).

Our service quality results, calculated using the Feature Tokenizer Transformer model, are shown in Fig 3. It can be seen that the service quality scores for different types of leisure consumption spaces generally range from 0.5 to 0.6, indicating relatively balanced development levels. Examining specific cases, eastern coastal cities and most provincial capitals show generally higher service quality, particularly Beijing, Shanghai, and Hangzhou. These cities demonstrate clear advantages in catering and entertainment leisure spaces. Conversely, western and southern regions exhibit relatively lower service quality levels, especially in personal care and sports facilities. In these less developed western and southern areas, slower urbanization processes result in weaker infrastructure and public service provision, along with less diversified consumer markets and demand variations, leading to generally inferior service quality in their leisure consumption spaces [70].

Comparing service quality across different types of leisure consumption spaces reveals that catering and entertainment show similar spatial patterns with comparable service levels, primarily concentrated in first-tier cities and eastern coastal areas. Catering establishments demonstrate relatively balanced service quality, particularly in high-frequency consumption areas with higher resident incomes. Entertainment spaces similarly achieve better service quality in these regions, mainly due to more diversified lifestyles and stronger entertainment demand. Retail and personal care leisure spaces exhibit more balanced nationwide service quality, though generally lower than catering and entertainment. Particularly in some second-tier cities and provincial capitals, retail malls and personal care facilities achieve limited operational effectiveness despite their scale, resulting in relatively poorer service quality. Finally, sports spaces show the lowest overall service scores with dispersed spatial distribution, indicating room for improvement in residents’ health awareness.

### 5.2. Residents’ life satisfaction in leisure consumption spaces

**Fig 4:**
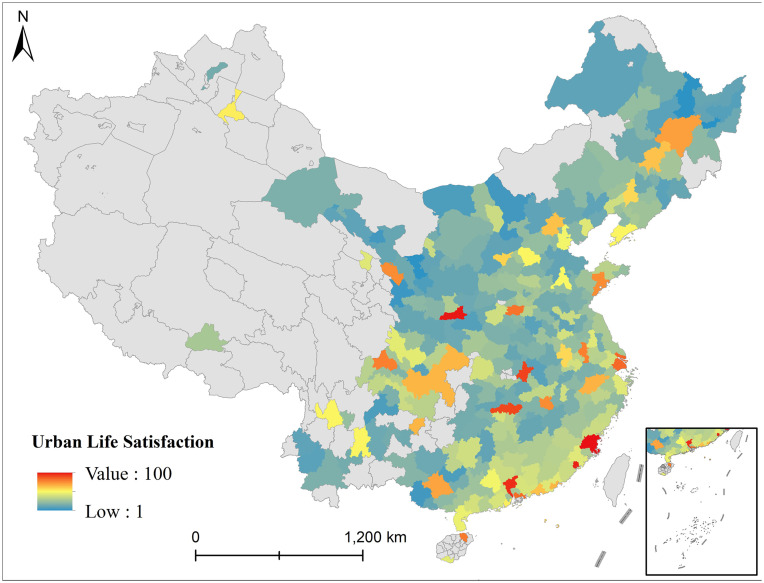
Residents’ life satisfaction in leisure consumption spaces. (This figure shows the national spatial pattern of residents’ life satisfaction in leisure consumption spaces. Specifically, it obtains this pattern based on RoBERTa sentiment analysis. Furthermore, it adopts a satisfaction rating scale of 1 to 100. In addition, it characterizes the level of life satisfaction in different cities with color depths or numerical gradients).

Using Weibo sign-in data and RoBERTa sentiment analysis, we calculate life satisfaction scores for leisure consumption spaces as shown in Fig 4. The results reveal a distinct spatial pattern – higher satisfaction in southeastern and coastal areas, lower in northwestern and inland regions, a distribution validated by other research data. Cities like Guangzhou, Shenzhen, Xiamen, Fuzhou, Hangzhou and Shanghai show dense clusters of positive sentiment posts, with frequent keywords like “clean”, “comfortable”, “delicious” and “good service”, mainly describing high-quality catering, entertainment and retail experiences. Wuhan, Changsha and Nanchang exhibit fewer negative emotions, with discussions focusing on “value-for-money” and “urban convenience” leisure scenarios. Northeastern cities (Harbin, Changchun, Shenyang) demonstrate low sign-in activity， with neutral/negative expressions like “deserted”, “poor service”, “boring” and “outdated”, reflecting declining vitality and user experience in leisure spaces. Northwestern regions (Lanzhou, Yinchuan, Xining) show poor spatial accessibility and limited leisure options, with frequent complaints like “not worth visiting”, “few choices” and “bad experience”, indicating dissatisfaction with public services and living convenience. The data shows that users in high-satisfaction areas more willingly combine consumption behaviors with social expression, preferring to leave positive reviews at spaces with good experiences. In contrast, users in low-satisfaction areas primarily sign in to record locations or provide functional information, with texts lacking emotional fluctuations. This demonstrates that urban residents’ leisure consumption behaviors closely correlate with their perception of space satisfaction [71].

### 5.3. Impact of different leisure consumption space development levels on life satisfaction

**Fig 5:**
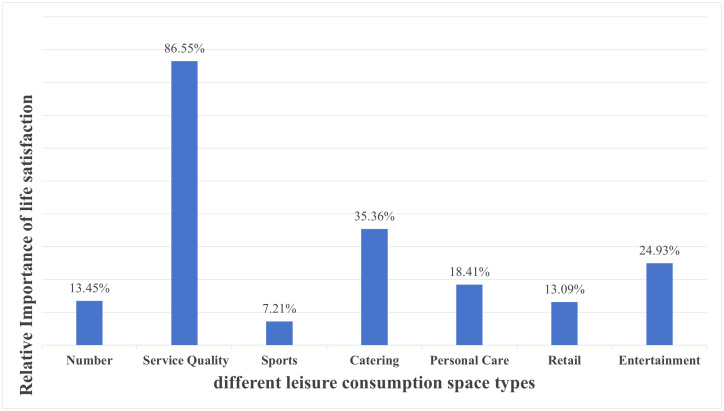
Relative importance of different leisure consumption spaces on life satisfaction. (This figure uses a combination form of bar charts and numerical tables. Furthermore, it accurately quantifies and shows the relative importance proportion of various influencing factors of leisure consumption spaces on residents’ life satisfaction. Specifically, its core contains two major dimensions. Firstly, the first dimension is the overall impact comparison between the facility quantity and service quality of leisure consumption spaces. Secondly, the second dimension is the subdivision impact comparison of the service quality among five types of spaces: dining, entertainment, healthcare, shopping, and sports. In addition, this figure presents all indicators in percentage form. Ultimately, they total 100%).

Fig 5 shows the relative importance results calculated by the model. Service quality accounts for 86.55% of the impact on life satisfaction, while the quantity of leisure consumption facilities contributes only 13.45%. This indicates that providing high-quality services improves residents’ life satisfaction more effectively than simply increasing the number of facilities.

The relative importance analysis of different urban leisure consumption spaces reveals that Catering space service quality has the strongest impact (36.36%) on life satisfaction, followed by Entertainment spaces (24.93%), Personal care spaces (18.41%), Retail spaces (13.09%), with Sports spaces showing the weakest influence (7.21%). Catering spaces meet both material needs and important social needs. These dining venues serve as spaces for communication, entertainment and social interaction, explaining their substantial impact on life satisfaction. Entertainment leisure encompasses not only traditional forms but also social gatherings and cultural activities. Improved service quality in entertainment spaces satisfies residents’ multi-level needs, producing notable effects on life satisfaction. While demand for personal care spaces grows in developed regions with increasing health awareness, their user base remains relatively smaller compared to catering and entertainment. Retail spaces show weaker impacts on life satisfaction, indicating shopping primarily fulfills material rather than emotional or psychological needs. Although shopping represents a common leisure activity, its functional nature limits its satisfaction-enhancing effects. Sports space service quality shows the weakest impact on life satisfaction, reflecting that sports activities depend more on individual health needs and exercise habits. Significant variations exist in residents’ sports requirements, and the lack of sports facilities in some areas prevents this type of leisure consumption space from broadly influencing residents’ life satisfaction.

Overall, the impact of service quality on life satisfaction reveals that people prioritize experiential needs over quantity in leisure consumption activities. While more leisure facilities provide options, they cannot enhance actual satisfaction if service quality fails to meet expectations. Today’s consumers increasingly seek high-quality services to fulfill emotional and psychological needs. This makes service quality in Catering and Entertainment spaces particularly influential on life satisfaction. The satisfaction stems not just from material fulfillment but also from psychological and emotional needs during consumption experiences, which directly affect life satisfaction ratings.

## 6. Discussion

We conduct a comprehensive analysis of leisure consumption space service quality and life satisfaction in major Chinese cities, examining the complex impact of service quality on residents’ life satisfaction. The study reveals differentiated effects of service quality across various leisure consumption space types on residents’ life satisfaction. Moreover, it verifies the core impact value of service quality on life satisfaction compared to facility quantity. Furthermore, the conclusions of this study confirm, supplement, and expand existing related research. At the same time, it combines interdisciplinary perspectives. Specifically, these perspectives include sociology, cultural background, and urban planning. Thus, it can provide a systematic internal mechanism explanation for the impact differences of different types of leisure consumption spaces. Additionally, it further clarifies the connection logic between leisure consumption spaces and residents’ life satisfaction. Ultimately, it also provides a deeper theoretical reference for follow-up research and urban practice.

Existing studies mainly focus on the quantity and spatial distribution of leisure consumption spaces, suggesting that denser distribution leads to better accessibility and higher development levels [72]. These studies tend to equate quantity and spatial density with development quality [73,74]. While quantity and distribution do affect service quality in some contexts, our analysis combining Dianping data with quantity distribution and service ratings shows denser leisure space distribution doesn’t necessarily mean better service quality [75]. This phenomenon appears particularly evident in less developed regions, revealing a disconnect between quantity distribution and actual consumption experience [43,76]. Therefore, leisure consumption space development depends not just on quantity and spatial density, but also on facility services, space functionality, and individual resident needs – findings that differ from existing research conclusions [77]. Existing studies typically assume quantity and distribution directly determine development level, while our analysis of actual rating data reveals the complex relationship between service quality and development level. This finding provides a new perspective for future research on leisure consumption spaces – spatial development should focus on space users rather than just quantity and spatial distribution.

In life satisfaction research, many scholars use traditional quantitative indicators to assess residents’ quality of life [78]. These indicators typically rely on questionnaire surveys or statistical data, but survey data often suffer from subjectivity and limitations due to sample selection and survey methods [79,80]. In contrast, our study analyzes sentiment in Weibo sign-in texts, capturing residents’ genuine emotional feedback about leisure consumption spaces more objectively and in real-time, avoiding biases inherent in traditional survey approaches. Existing studies using Weibo sign-in data for urban space research typically focus on single space types or specific themes, analyzing large-scale text data to examine overall space quality or trends [81,82]. Our study targets leisure consumption spaces, comparing subjective perceptions across different types to reveal more nuanced variations in residents’ life satisfaction. For instance, some retail areas show high sign-in volumes but low service quality or environmental conditions in sentiment analysis – findings that might remain undiscovered in previous studies.

In the field of life satisfaction research, many scholars evaluate residents’ quality of life based on traditional quantitative indicators such as housing conditions and transportation convenience. Furthermore, these indicators mostly rely on questionnaire surveys or statistical data. Therefore, sample selection and survey methods easily affect them. As a result, they have subjectivity and limitations. However, this study conducts sentiment analysis on Weibo sign-in data texts. Through this, it captures residents’ real emotional feedback on leisure consumption spaces more objectively and in real time. Consequently, it makes up for the deviations of traditional survey methods. Currently, existing urban space studies use Weibo sign-in data. However, they mostly limit themselves to a certain type of space or a specific theme. Thus, they explore the overall quality or trends of spaces through large-scale text data analysis. In contrast, this study takes leisure consumption spaces as the overall research object. Furthermore, it comparatively analyzes the subjective feeling differences among different types of spaces. Consequently, it more meticulously reveals the differences in residents’ life satisfaction across various leisure consumption spaces. For example, shopping spaces in some areas have a large volume of sign-in data. However, sentiment analysis shows that their service quality and environmental evaluations are low. Previously, single-dimension studies hardly reflect this detail. Besides, existing studies often explore the relationship between the development of leisure consumption spaces and life satisfaction through spatial distribution density. However, they do not fully consider service quality and individual demand differences. In contrast, this study conducts a combined analysis of Dianping service scores and spatial distribution. Through this, it confirms that some cities have many leisure consumption spaces. However, low service quality evaluations lead to no significant improvement in life satisfaction. Furthermore, the quantity distribution of leisure consumption spaces and service quality do not have a simple directly proportional relationship. In addition, underdeveloped regions still face the problem of a disconnection between service quality and residents’ actual demands.

Existing studies generally agree that the diversity of urban leisure consumption spaces significantly affects residents’ life satisfaction, with different space types meeting diverse material, emotional and health needs [48]. Research shows Catering, Entertainment and Sports spaces have varying impacts on life satisfaction [83]. However, these studies often focus on single space types, neglecting the multidimensional effects of service quality across different leisure spaces [29]. Our study reveals the relative importance of service quality, demonstrating the dominant role of Catering and Entertainment spaces – a finding consistent with experience-oriented consumption theory, where residents prioritize participation over mere accessibility and quantity [84]. Notably, we find service quality outweighs facility quantity as the core determinant of life satisfaction, indicating a shift in usage value from availability to perceived quality, which crucially supports modern urban residents’ happiness construction [85].

From an interdisciplinary perspective, multiple factors jointly cause the impact differences of different types of leisure consumption spaces on life satisfaction. Specifically, these factors include cultural characteristics, social relationship construction, and urban planning layout. Consequently, their internal mechanisms have distinct comprehensive characteristics. From the perspective of cultural background, China’s profound “food culture” makes dining exceed the mere satisfaction of physiological needs. Thus, it becomes the core carrier for emotional expression and relationship maintenance. Consequently, this makes dining spaces a high-frequency and strongly associated leisure choice for residents. Furthermore, the experiential perception of their service quality directly affects daily subjective well-being. Therefore, dining spaces also become the most significantly impactful type among various spaces. Meanwhile, Chinese residents possess a long-formed “static leisure” life culture. This culture helps residents accept low-participation-threshold leisure methods, such as entertainment and shopping, more easily. By contrast, society does not fully form the cultural atmosphere of sports-based dynamic leisure yet. As a result, this weakens its impact [86]. From a sociological perspective, leisure consumption spaces are important fields for social relationship construction and social capital accumulation. Furthermore, dining and entertainment spaces serve as core scenes for residents’ primary social relationship connections and secondary social relationship expansion. Their high-quality service experience can enhance the pleasure of social interactions. Moreover, it strengthens residents’ sense of belonging and community identity. In contrast, shopping spaces mostly focus on instrumental consumption. Thus, they lack strong social attributes. Meanwhile, health care and sports spaces have stratified and personalized audience groups. Because of this, they hardly form broad social relationship connections. Therefore, their impact degrees on overall life satisfaction decrease sequentially. At the same time, the stratified characteristics of residents’ consumption demands further amplify this difference. Specifically, health care and sports spaces have relatively higher consumption thresholds. Consequently, their audience coverage range is far lower than that of dining and entertainment spaces. Accordingly, the universality of their impact also weakens [87]. From an urban planning perspective, the layout of urban leisure consumption spaces in China shows an obvious characteristic of “priority embedding of dining and entertainment”. Specifically, these types of spaces mainly exist in residents’ daily life circles. Thus, their accessibility and usage convenience are far higher than those of health care, sports, and some shopping spaces. In contrast, health care and sports spaces often concentrate in specific urban areas or new urban districts. Consequently, they lack community-level supply and have low accessibility. Meanwhile, shopping spaces face the problem of a severe homogenized layout. Furthermore, they lack experiential and characteristic designs. Ultimately, the layout differences and supply imbalances of urban planning further strengthen the impact hierarchy of different types of leisure consumption spaces on life satisfaction [88].

This study explores the impact differences of leisure consumption spaces on residents’ life satisfaction based on social media big data. Thus, it provides a new paradigm of big data analysis for research in this field. Moreover, from the perspective of research sustainability and subsequent expansion, future research can further deepen this topic. Specifically, it can combine the green development, industrial integration innovation, and smart service directions of leisure consumption spaces. Additionally, the research results in related fields also provide important references for the extension of this research. The sustainable development of leisure consumption spaces, as a core component of urban service scenes, draws upon research findings in the green development of the hotel industry. It integrates the concepts of green transformational leadership and green human resource management into the operation and management of these spaces. Through concept guidance and talent cultivation, it promotes green innovation in dining, entertainment, and accommodation leisure spaces, creates low-carbon ecological leisure consumption scenes, and thus enriches the dimensions through which leisure consumption spaces influence life satisfaction [89]. Meanwhile, leisure consumption is highly integrated with the tourism industry. Therefore, the research approach of achieving sustainable tourism through innovation and knowledge is applicable to the study of urban leisure consumption spaces. Relying on knowledge and technological innovation, it drives the upgrade of service models and product forms in leisure consumption spaces, thereby achieving their connotative sustainable development [90]. In addition, smart service technologies such as the hotel check-in system based on face recognition provide a technical approach for the intelligent upgrading of leisure and consumption spaces. The application of technologies including face recognition and intelligent terminals to the service process of leisure and consumption spaces can achieve intelligent, personalized and efficient services and further improve service quality. Moreover, the impact of smart service quality on residents’ life satisfaction will also become an important expansion direction of research in this field from the perspective of social media big data [91].

Through systematic analysis of how urban leisure consumption space service quality affects life satisfaction, our study makes two key contributions. First, we combine Dianping review data and Weibo sign-in data to respectively reflect service quality and subjective emotional experiences, establishing a relationship chain between leisure spaces and satisfaction from both material and human perspectives. Second, by employing deep learning sentiment classification models and service rating models, we quantify and spatially map subjective perceptions alongside objective quality measures, achieving effective coupling between physical locations, service provision and user perception.

Based on empirical findings regarding the relationship between leisure consumption space service quality and residents’ life satisfaction, this study proposes targeted practical recommendations in urban planning and public service strategies. These evidence-based suggestions account for varying urban development phases and spatial characteristics, with the dual objectives of improving policy relevance and enhancing residents’ perceived well-being.

Urban spatial planning policies should prioritize context-specific allocation strategies tailored to regional characteristics. In eastern coastal cities, the emphasis should shift from quantitative expansion to qualitative refinement—optimizing service delivery in existing dining and entertainment venues to prevent homogeneous facility clustering, while strategically expanding targeted health and sports facilities. Conversely, western and less developed cities require fundamental service functionality improvements in basic leisure infrastructure rather than mere numerical expansion. Aligning with local demographic profiles, these cities should develop cost-effective, highly accessible leisure spaces near aging urban districts to address dual challenges of spatial inaccessibility and limited recreational variety. Public service strategies should establish robust quality assurance mechanisms through two key approaches. First, an integrated service quality evaluation framework for leisure consumption spaces should be implemented, incorporating multi-source data from platforms like Dianping ratings and Weibo sentiment analysis into regular monitoring cycles, with targeted interventions for persistently underperforming facilities. Second, differentiated service policies must address demographic disparities—specifically enhancing public leisure spaces with age-appropriate facilities and budget-friendly options for elderly and low-income residents, thereby compensating for the current data gap in representing these vulnerable populations.

## 7. Conclusion

This study evaluates service quality of urban leisure consumption spaces in major Chinese cities using the FT-Transformer model based on Dianping data. Combined with Weibo sign-in data, we further analyze residents’ life satisfaction in these spaces using the RoBERTa model. Finally, we analyze the relative importance of different urban leisure consumption space development levels on life satisfaction using the XGBoost model. The main findings show that: first, high-quality urban leisure consumption spaces significantly enhance residents’ life satisfaction. Compared to the quantity of leisure consumption spaces, their superior service quality not only fulfills material needs but also strengthens psychological security and sense of belonging toward the urban environment. Second, different types of leisure consumption spaces show varying impacts on residents’ life satisfaction. Catering and entertainment spaces demonstrate the most significant positive effects. Retail spaces show relatively weaker impacts – while retail represents a common leisure activity, it primarily satisfies material rather than emotional or social needs. Personal care and sports spaces exhibit regional variations in their effects on life satisfaction, though their overall influence remains comparatively modest. Third, this study combines Dianping data and Weibo sign-in data to conduct a multi-dimensional evaluation of the relationship between leisure consumption spaces and residents’ life satisfaction. Dianping data and Weibo sign-in data reflect consumers’ actual experiences and emotional feedback, further revealing residents’ emotional tendencies and subjective satisfaction across different leisure spaces. Such multi-dimensional evaluation method based on social media and online review platforms overcomes potential biases in traditional questionnaire surveys. It captures residents’ genuine emotions and spatial usage behaviors in real-time, providing new perspectives and data support for urban spatial research.

Finally, we identify several limitations in our study. First, while Dianping and Weibo data are extensive, their user base primarily consists of younger and middle-to-high income groups, potentially underrepresenting elderly and low-income populations’ perceptions and needs. Second, the current study mainly reveals the relative importance of impacts while lacking exploration of mediating variables or process mechanisms in causal chains. Considering the limitations of this study and the development trends in the field, subsequent research can deepen the investigation from three aspects. First, future studies can enrich the research dimensions and data sources by incorporating dimensions such as green development and smart services. They can integrate offline survey data to compensate for the population coverage limitations of social media big data, while also conducting heterogeneity analysis for different age and income groups. Second, further research can delve into the influence mechanisms and dynamic characteristics by introducing mediating and moderating effects to explore the transmission pathways through which leisure consumption spaces affect life satisfaction. It can also employ panel data for longitudinal studies to reveal the dynamic evolution patterns of this relationship. Third, the research scope and practical boundaries can be expanded by conducting comparative studies of urban and rural leisure consumption spaces. This includes focusing on scenarios such as urban renewal and urban agglomeration development to analyze the impact of the restructuring of leisure space layout on resident satisfaction. Additionally, by integrating theories such as green transformational leadership and sustainable tourism innovation, it can explore practical pathways for the high-quality development of leisure consumption spaces.
